# Identification of the *WRKY* gene family and functional analysis of two genes in *Caragana intermedia*

**DOI:** 10.1186/s12870-018-1235-3

**Published:** 2018-02-09

**Authors:** Yongqing Wan, Mingzhu Mao, Dongli Wan, Qi Yang, Feiyun Yang, Mandlaa, Guojing Li, Ruigang Wang

**Affiliations:** 1 College of Life Sciences, Inner Mongolia Key Laboratory of Plant Stress Physiology and Molecular Biology, Inner Mongolia Agricultural University, Hohhot, China; 2 Institute of Grassland Research, Chinese Academy of Agricultural Sciences, Key Laboratory of Grassland Ecology and Restoration, Ministry of Agriculture, Hohhot, China; 3 College of Food Science and Engineering, Inner Mongolia Agricultural University, Hohhot, China

**Keywords:** WRKY gene family, *Caragana Intermedia*, Abiotic stress, Gene expression, Subcellular localization, Function analysis

## Abstract

**Background:**

WRKY transcription factors, one of the largest families of transcriptional regulators in plants, play important roles in plant development and various stress responses. The WRKYs of *Caragana intermedia* are still not well characterized, although many WRKYs have been identified in various plant species.

**Results:**

We identified 53 *CiWRKY* genes from *C. intermedia* transcriptome data, 28 of which exhibited complete open reading frames (ORFs). These CiWRKYs were divided into three groups via phylogenetic analysis according to their WRKY domains and zinc finger motifs. Conserved domain analysis showed that the CiWRKY proteins contain a highly conserved WRKYGQK motif and two variant motifs (WRKYGKK and WKKYEEK). The subcellular localization of CiWRKY26 and CiWRKY28–1 indicated that these two proteins localized exclusively to nuclei, supporting their role as transcription factors. The expression patterns of the 28 *CiWRKYs* with complete ORFs were examined through quantitative real-time PCR (qRT-PCR) in various tissues and under different abiotic stresses (drought, cold, salt, high-pH and abscisic acid (ABA)). The results showed that each *CiWRKY* responded to at least one stress treatment. Furthermore, overexpression of *CiWRKY75–1* and *CiWRKY40–4* in *Arabidopsis thaliana* suppressed the drought stress tolerance of the plants and delayed leaf senescence, respectively.

**Conclusions:**

Fifty-three *CiWRKY* genes from the *C. intermedia* transcriptome were identified and divided into three groups via phylogenetic analysis. The expression patterns of the 28 *CiWRKYs* under different abiotic stresses suggested that each Ci*WRKY* responded to at least one stress treatment. Overexpression of *CiWRKY75–1* and *CiWRKY40–4* suppressed the drought stress tolerance of *Arabidopsis* and delayed leaf senescence, respectively. These results provide a basis for the molecular mechanism through which CiWRKYs mediate stress tolerance.

**Electronic supplementary material:**

The online version of this article (10.1186/s12870-018-1235-3) contains supplementary material, which is available to authorized users.

## Background

Transcription factors are a class of proteins that significantly regulate plant growth and development and play an important role in plant defense and stress responses [1, 2]. WRKY transcription factors are one of the largest families of transcriptional regulators in plants [2]. These transcription factors contain the WRKY domain, which is a region of 60 amino acids that is highly conserved [3]. The WRKY domain comprises a featured WRKYGQK sequence motif and a zinc finger-like motif [4]. The WRKY sequence is occasionally replaced by WRRY, WSKY, WKRY, WVKY or WKKY in a subset of WRKY proteins [5]. The zinc finger structure is either CX_4–5_CX_22–23_HXH (C_2_H_2_-type) or CX_7_CX_23_HXC (C_2_HC-type) [5]. At least one WRKY domain is present in all known WRKY proteins [3].

WRKY proteins can be classified into three main groups (group I, group II and group III) according to the number of WRKY domains along with features of the zinc finger-like motif [3]. Group I proteins have two WRKY domains: an N-terminal WRKY domain and a C-terminal WRKY domain, with C_2_H_2_ zinc finger type CX_4_CX_22-23_HXH. Group II proteins harbor a single WRKY domain and a C_2_H_2_-type CX_4–5_CX_23_HXH zinc finger. Group III proteins contain a single WRKY domain and a C_2_HC-type CX_7_CX_23_HXC zinc finger [3]. Group II WRKY proteins were further divided into five subgroups on the basis of their primary amino acid sequences: IIa, IIb, IIc, IId and IIe [5]. Furthermore, WRKY proteins specifically bind to the W box (C/T)TGAC(T/C) DNA sequence [3, 4], and this binding requires both WRKY and zinc finger domains [4].

The first 4 *WRKY* cDNAs (*SPF1*, *ABF* (*1*, *2*), *PcWRKY* (*1*, *2*, *3*) and *ZAP1*) were cloned from sweet potato (*Ipomoea batatas*), wild oat (*Avena fatua*), parsley (*Petroselinum crispum*) and *Arabidopsis thaliana*, respectively [3]. Numerous WRKY proteins have been studied in various plant species. For example, there are 72 identified members in *A. thaliana* [6], 182 in soybean (*Glycine max*) [7], 127 in apple (*Malus domestica Borkh.*) [8], 80 in grape (*Vitis vinifera*) [9], 86 in *Brachypodium distachyon* [10], 85 in cassava (*Manihot esculenta*) [11], 95 in carrot (*Daucus carota*) [12], 61 in *Salvia miltiorrhiza* [1], 103 in rice (*Oryza sativa*) [13], and 32 in Broomcorn millet (*Panicum miliaceum L.*) [14]. In maize (*Zea mays*), 136 WRKY proteins encoded by 119 *WRKY* genes have been identified [15]. In cotton, 116 and 102 *WRKY* genes have been identified from *Gossypium raimondii* and *Gossypium hirsutum* [16], respectively.

WRKY transcription factors are involved in multiple plant processes, such as seed development [17, 18], dormancy [19] and germination [20], trichome development [21], root development [22], leaf senescence [23], plant growth and flowering [24, 25]. Notably, WRKYs have been broadly reported to be involved in plant defense responses. For instance, 49 out of 72 *WRKYs* from *A. thaliana* are differentially expressed after pathogen infection or SA treatment [6], and *A. thaliana* WRKYs including AtWRKY7, 11, 17, 18, 23, 25, 27, 38, 40, 41, 48, 53, 58, 60, and 62 serve as negative regulators of defense signaling [26]. OsWRKY62 and OsWRKY76 of rice negatively regulate defense upon pathogen infection [27]. Overexpression of *OsWRKY13* [28] and *OsWRKY45–2* [29] in rice increases resistance to bacterial blight and fungal blast caused by *Xanthomonas oryzae* pv. *Oryzae* and *Magnaporthe grisea*, respectively, while overexpression of *OsWRKY42* decreases rice resistance to *M. oryzae* [30]. The transcriptional regulatory cascade of WRKY13, WRKY42, and WRKY45–2 is necessary for defending rice against fungal pathogens [30]. In cotton (*G. hirsutum L*.), GhWRKY15 is involved in disease resistance [31]. Overexpression of grape *VvWRKY1* in tobacco results in enhanced resistance to various fungi [32]. The roles of WRKYs in plant responses to abiotic stresses such as drought [33–36], cold [37], salinity [37], heat [38, 39], low Pi [40, 41], ultraviolet B (UV-B) [42], osmotic stress [43], and ABA [33, 36, 44] have been widely reported. These studies suggested that WRKYs are involved in multiple stress responses.

*Caragana intermedia*, a native desert perennial shrub belonging to the Leguminosae family, is distributed in sandy grasslands and desert regions of west and northwest China and Mongolia, and is widely valued due to its high forage value and sand-fixing capacity, along with its strong resistance to drought and salinity [45–48]. *C. intermedia* is considered to be an ideal plant for elucidating the mechanisms of abiotic stress tolerance [49], and research on its molecular mechanisms has been increasing in recent years. For instance, to normalize quantitative real-time PCR (qRT-PCR) data from *C. intermedia* under different abiotic stress conditions, suitable reference genes were screened, which was useful for further gene expression analysis [50]. The transcription of two NAC transcription factors, *CiNAC3* and *CiNAC4*, was found to be induced under treatment with ABA and various abiotic stresses, and ectopic expression of *CiNAC3* and *CiNAC4* in *Arabidopsis* reduces the inhibition of seed germination by ABA and enhances the salt tolerance of the transgenic plants [51]. The expression patterns of miR2118 and its target genes (*CiDR1* and *CiDR2*) from *C. intermedia* are altered under drought stress, and constitutive overexpression of cin-miR2118 in tobacco enhances the plant’s tolerance to drought stress [46]. Abiotic stresses (NaCl, ZnSO_4_, CdCl_2_, high/low temperature, and dehydration) induce the expression of the glutamate decarboxylase-encoding genes *CiGAD1* and *CiGAD2*, except for *CiGAD2* under Cd stress, and ABA has been shown to be involved in regulating the expression of *CiGADs* in response to salt stress [52]. However, there is limited knowledge of the function of WRKYs in *C. intermedia*. In this study, we identified 53 *CiWRKY* genes based on transcriptomic data from *C. intermedia*, performed phylogenetic analysis and WRKY domain alignment, and examined the subcellular localizations of two *CiWRKYs* and the expression patterns of 28 of *CiWRKYs* in different tissues and under various abiotic stresses. Furthermore, we overexpressed *CiWRKY75–1* and *CiWRKY40–4* in *A. thaliana* and characterized their function. This work provides a basis for exploring the molecular roles of *WRKYs* and facilitates further investigation of the molecular mechanisms of abiotic stress tolerance in *C. intermedia.*

## Results

### Identification and cloning *WRKY* genes from *C. intermedia*

To identify *WRKYs* in the drought-treated transcriptome of *C. intermedia*, the sequences of the *A. thaliana* WRKY family were used to query homologous *C. intermedia* sequences. After Blast searches against the sequences in NCBI using blastX to predict conserved domains and remove redundant sequences, 53 sequences with apparent WRKY domains were annotated as *C. intermedia WRKY* genes. Among these genes, 21 *WRKYs* with complete open reading frames (ORFs) were identified. Using the rapid-amplification of *cDNA* ends (RACE) technique, an additional 7 *WRKY*s were cloned, and their full-length ORFs were obtained. The ORF lengths of these 28 genes ranged from 489 bp to 2229 bp, and their amino acid lengths ranged from 163 to 743 aa (details are provided in Table 1). The 28 *WRKY*s with complete ORFs were used for further expression analysis.

**Table 1:** Characteristics of *WRKY* genes in *C. intermedia*

Gene name	cDNA length	Aa	Mw (KDa)	Group	WRKY domain	Zinc finger	*Arabidopsis* ortholog
*CiWRKY2*	2229	743	80.46	Group I	WRKYGQK×2	CX_4_CX_22-23_HXH	*AtWRKY2*
*CiWRKY3–1*	1383	461	50.31	Group I	WRKYGQK×2	CX_4_CX_22-23_HXH	*AtWRKY3*
*CiWRKY3–2*	1536	512	55.86	Group I	WRKYGQK×2	CX_4_CX_22-23_HXH	*AtWRKY3*
*CiWRKY6–1*	1656	552	59.97	Group IIb	WRKYGQK	CX_5_CX_23_HXH	*AtWRKY6*
*CiWRKY6–2*	1830	610	65.56	Group IIb	WRKYGQK	CX_5_CX_23_HXH	*AtWRKY6*
*CiWRKY12*	696	232	26.32	Group IIc	WRKYGQK	CX_4_CX_23_HXH	*AtWRKY12*
*CiWRKY15*	1005	335	36.43	Group IId	WRKYGQK	CX_5_CX_23_HXH	*AtWRKY15*
*CiWRKY17*	1017	339	36.59	Group IId	WRKYGQK	CX_5_CX_23_HXH	*AtWRKY17*
*CiWRKY23*	957	319	35.71	Group IIc	WRKYGQK	CX_4_CX_23_HXH	*AtWRKY23*
*CiWRKY26*	1722	574	63.28	Group I	WRKYGQK×2	CX_4_CX_22-23_HXH	*AtWRKY26*
*CiWRKY28–1*	999	333	36.98	Group IIc	WRKYGKK	CX_4_CX_23_HXH	*AtWRKY28*
*CiWRKY28–2*	975	325	37.19	Group IIc	WRKYGQK	CX_4_CX_23_HXH	*AtWRKY28*
*CiWRKY30*	1083	361	40.79	Group III	WRKYGQK	CX_7_CX_23_HXC	*AtWRKY30*
*CiWRKY32–1*	1521	507	54.68	Group I	WRKYGQK×2	CX_4_CX_22-23_HXH	*AtWRKY32*
*CiWRKY33–1*	1614	538	59.57	Group I	WRKYGQK×2	CX_4_CX_23_HXH	*AtWRKY33*
*CiWRKY36*	1785	595	64.36	Group IIb	WRKYGQK	CX_5_CX_23_HXH	*AtWRKY36*
*CiWRKY40–1*	990	330	36.78	Group IIa	WRKYGQK	CX_5_CX_23_HXH	*AtWRKY40*
*CiWRKY40–3*	825	275	30.97	Group IIa	WRKYGQK	CX_5_CX_23_HXH	*AtWRKY40*
*CiWRKY40–4*	897	299	33.66	Group IIa	WRKYGQK	CX_5_CX_23_HXH	*AtWRKY40*
*CiWRKY41–1*	1083	361	41.12	Group III	WRKYGQK	CX_7_CX_23_HXC	*AtWRKY41*
*CiWRKY48*	1155	385	42.15	Group IIc	WRKYGQK	CX_4_CX_23_HXH	*AtWRKY48*
*CiWRKY50*	489	163	19.22	Group IIc	WRKYGKK	CX_4_CX_23_HXH	*AtWRKY50*
*CiWRKY51*	558	186	21.55	Group IIc	WRKYGKK	CX_4_CX_23_HXH	*AtWRKY51*
*CiWRKY56*	678	226	25.61	Group IIc	WRKYGQK	CX_4_CX_23_HXH	*AtWRKY56*
*CiWRKY57*	834	278	30.68	Group IIc	WRKYGQK	CX_4_CX_23_HXH	*AtWRKY57*
*CiWRKY69–1*	798	266	28.89	Group IIe	WRKYGQK	CX_5_CX_23_HXH	*AtWRKY69*
*CiWRKY70–1*	939	313	35.24	Group III	WRKYGQK	CX_7_CX_23_HXC	*AtWRKY70*
*CiWRKY75–1*	567	189	21.08	Group IIc	WRKYGQK	CX_4_CX_23_HXH	*AtWRKY75*

*Mw* Molecular weight, *Aa* amino acid length

### Phylogenetic analysis and multiple sequence alignment of WRKY domains

**Fig. 1:**
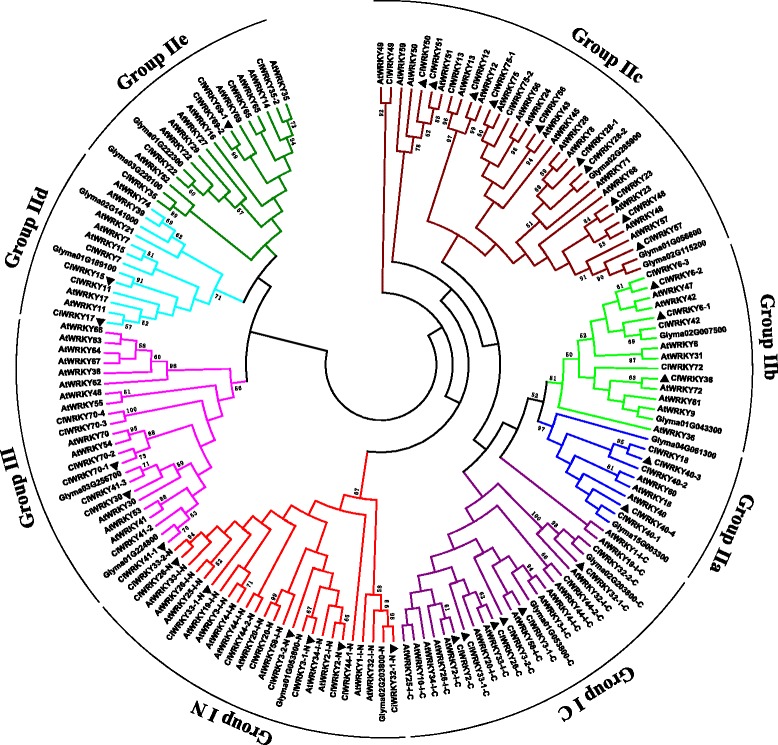
Phylogenetic tree of all WRKY proteins from *C. intermedia*, *A. thaliana* and *G. max.* ‘N’ and ‘C’ indicate the N-terminal and C-terminal WRKY domains of a specific WRKY protein, respectively. The neighbor-joining method was applied to construct the phylogenetic tree

To further analyze the evolution of these CiWRKYs, we constructed a phylogenetic tree based on a total of 140 WRKYs (72 from *A. thaliana*, 53 from *C. intermedia* and 15 from *G. max* distributed in different groups) (Fig. 1). According to the number of WRKY domains and the pattern of zinc finger structures, the 53 CiWRKY proteins were distributed in three main groups. Eleven CiWRKYs were assigned to group I. Thirty-four CiWRKYs were distributed in group II, which was further classified into five sub-groups: IIa, IIb, IIc, IId, and IIe, which contained 5, 6, 13, 4 and 6 CiWRKYs, respectively. The remaining 8 CiWRKYs belonged to group III.

**Fig. 2:**
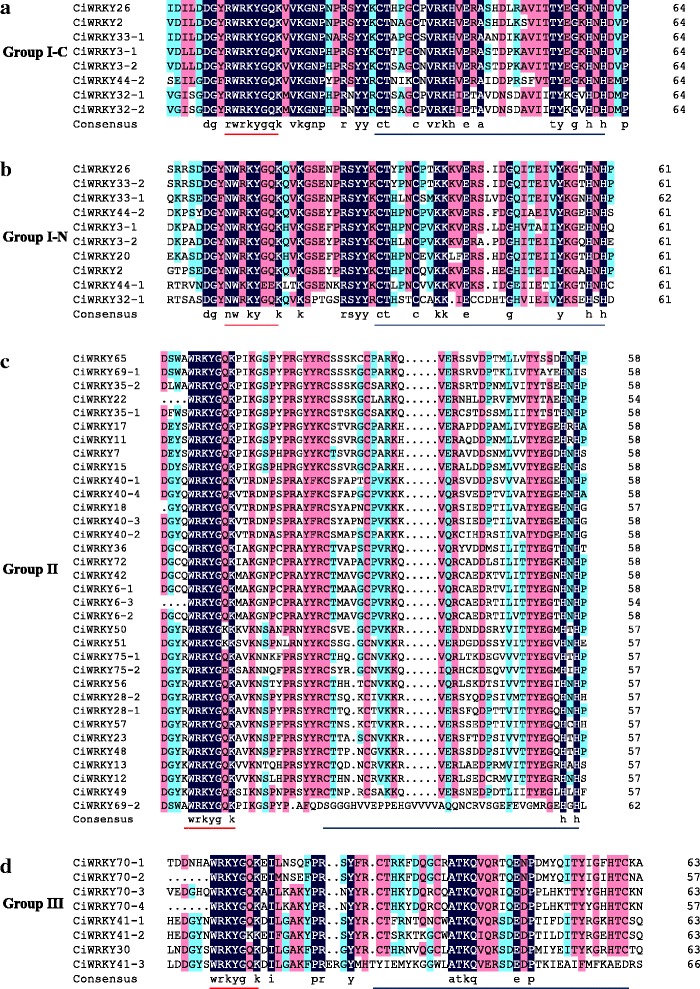
Multiple sequence alignments of the WRKY domains of from CiWRKYs. **a** C-terminal WRKY domains of group I proteins. **b** N-terminal WRKY domains of group I proteins. **c** WRKY domains of group II proteins. **d** WRKY domains of group III proteins. The conserved WRKY amino acid signature is indicated by red bars, and the zinc finger motif is indicated by black bars

Multiple sequence alignments of the core domains of the CiWRKYs showed that, in group I, with the exception of CiWRKY44–1, which exhibited a WKKYEEK variant sequence, all others harbored the conserved WRKYGQK sequence, and all displayed the apparent C_2_H_2_-typezinc finger (CX_4_CX_22-23_HXH). Among the 34 group II proteins containing one WRKY domain, 32 CiWRKYs carried the conserved WRKYGQK sequence, while CiWRKY50 and CiWRKY51 exhibited the WRKYGKK sequence instead. The C_2_H_2_-type zinc finger (CX_4–5_CX_23_HXH) was observed in 33 CiWRKYs. Seven of the eight group III WRKYs harbored the WRKYGQK sequence, while the variant sequence WRKYGKK was present in CiWRKY41–2. The other group III CiWRKYs exhibited C_2_HC-type zinc finger (CX_7_CX_23_HXC) with the exception of CiWRKY41–3 (Fig. 2).

### Subcellular localization of CiWRKYs

**Fig. 3:**
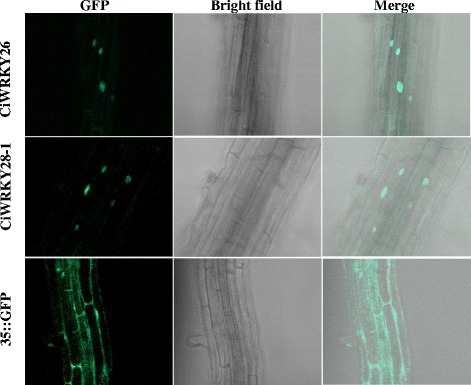
Subcellular localization of two *C. intermedia* WRKYs. The roots of *35S::CiWRKY26*-*GFP* (upper panel), *35S::CiWRKY28–1*-*GFP* (middle panel), and *35S::GFP* (bottom panel) transgenic plants were used for observations of GFP fluorescence

To examine the subcellular localization of the CiWRKY proteins, one of the proteins containing two WRKY domains (CiWRKY26) and one of the proteins containing one-WRKY-domain (CiWRKY28–1) were fused with green fluorescent protein (GFP). The roots of the T2 generation of *35S*::*CiWRKYs*-*GFP* transgenic *Arabidopsis* seedlings were used to examine GFP activity. GFP fluorescence was exclusively observed in the nuclei of the transgenic *Arabidopsis* seedlings, while GFP was ubiquitously present throughout the cells of the *35S::GFP* transgenic lines (Fig. 3). The nuclear localization of these two *CiWRKYs* is in agreement with their putative roles as transcription factors.

### Expression patterns of *CiWRKYs* in *C. intermedia*

**Fig. 4:**
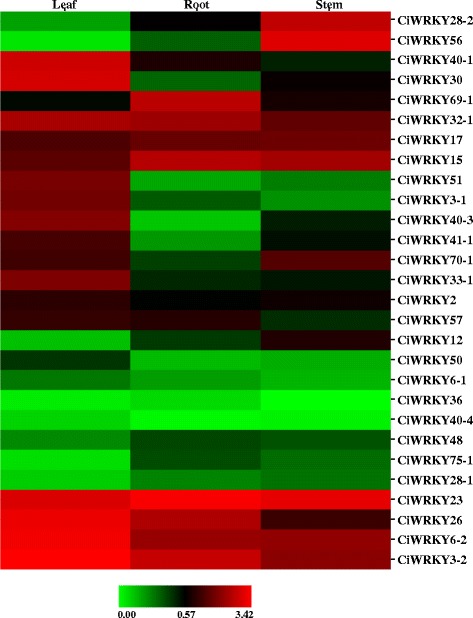
Expression profiles of *CiWRKY* genes in different tissues. Samples were collected from the roots (R), stems (S) and leaves (L) of *C. intermedia*. The transcripts of 28 *CiWRKYs* with full-length sequences were examined via qRT-PCR. The relative expression values were calculated using the 2^-ΔCT^ method, and *CiEF1α* was employed as the endogenous control. The 2^-ΔCT^-based expression values were used to produce the heatmap

The expression patterns of the 28 *CiWRKYs* with complete ORFs in wild *C. intermedia* tissues, including the roots, stems and leaves, were examined via qRT-PCR. As shown in Fig. 4 and Additional file 1: Table S1, four genes (*CiWRKY3–2*, *CiWRKY6–2*, *CiWRKY23* and *CiWRKY26*) exhibited relatively high expression levels in all examined tissues, especially in the leaves. *CiWRKY32–1*, *CiWRKY17* and *CiWRKY15* were also ubiquitously expressed, but at relatively low levels compared with the above four genes. In contrast, the transcripts of eight genes, including *CiWRKY36*, *CiWRKY75–1*, *CiWRKY12*, *CiWRKY50*, *CiWRKY28–1*, *CiWRKY48*, *CiWRKY40–4* and *CiWRKY6–1*, were detectable, but showed extremely low abundance in all tissues. *CiWRKY28–2* and *CiWRKY56* were mainly expressed in stems, while *CiWRKY40–1* and *CiWRKY30* were predominantly found in leaves. *CiWRKY69–1* showed higher transcript abundance in the roots than in other tissues. The other *CiWRKY* genes showed lower expression globally, and most of them exhibited lower transcript abundance in the roots than in the leaves or stems.

### Expression patterns of *CiWRKYs* under abiotic stresses

Malondialdehyde (MDA), proline (PRO) and soluble total sugar (SS) are indicators of plant physiological status. As shown in Additional file 2: Figure S1, drought treatment induced the accumulation of MDA, PRO and SS, whose levels peaked at 48 h. These findings indicated that the applied drought treatment was effective.

**Fig. 5:**
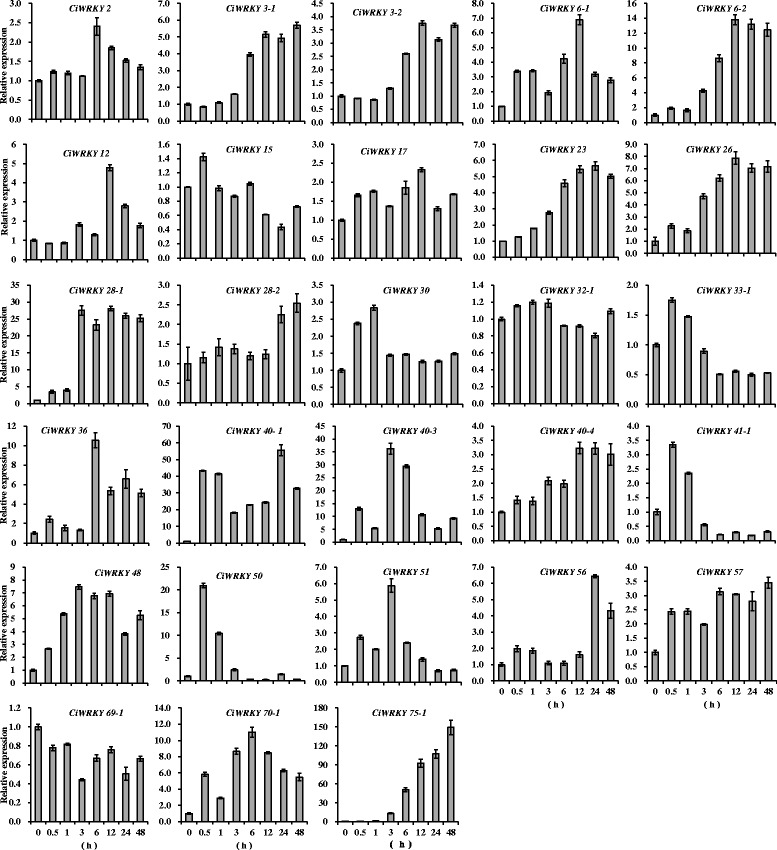
Expression patterns of *CiWRKYs* under drought treatment. Samples were collected from the shoots of one-month-old *C. intermedia* seedlings at 0.5, 1, 3, 6, 12, 24 or 48 h following drought treatment, and untreated plants were employed as the control. The expression levels of 28 *CiWRKYs* with full-length sequences were examined via qRT-PCR. Expression values were estimated using the 2^-ΔΔCT^ method, and *CiEF1α* was used as reference gene. The error bars represent the means of three technical replicates ± SD

The expression patterns of the 28 of *CiWRKYs* were further assessed using qRT-PCR, under drought treatment as well as other abiotic stresses, including salt, abscisic acid (ABA), cold, and high-pH treatments (Table 2). The gene expression of twenty-four *CiWRKYs* genes was found to be induced by drought treatment, with changes of 2- to 149-fold. In contrast, the expression of *CiWRKY33–1*, *CiWRKY15* and *CiWRKY69–1* was inhibited by drought treatment, and no obvious change in the expression of *CiWRKY32–1* was observed (Fig. 5). Salt treatment down-regulated the expression of 18 *CiWRKYs* and up-regulated the expression of 5 *CiWRKYs* (Additional file 3: Figure S2). Under ABA treatment, the expression of 22 *CiWRKYs* was increased, while that of *CiWRKY3–1* and *CiWRKY33–1* was decreased, and *CiWRKY2*, *3–2*, *32–1* and *69–1* were not obviously affected (Additional file 4: Figure S3). Under cold treatment, the expression of 14 *CiWRKYs* was up-regulated, and the transcripts of 11 *CiWRKYs* were down-regulated. The expression of three other *CiWRKYs* presented no change compared with the expression seen in the control (Additional file 5: Figure S4). High-pH treatment enhanced the expression of 12 *CiWRKYs*. Notably, the maximal expression observed for *CiWRKY33–1*, *41–1*, *50*, and *40–4* corresponded to increases of 111-, 365-, 388-, and 109-fold, respectively, whereas the expression of the other *CiWRKYs* was not affected or even down-regulated after high-pH treatment (Additional file 6: Figure S5). The results indicated that each of these *CiWRKY* genes responded to at least one stress treatment.

**Table 2:** Expression patterns of *CiWRKYs* under different abiotic stresses

Gene name	Drought	Salt	ABA	Cold	High-pH
*CiWRKY2*	up	N	N	down	up
*CiWRKY3–1*	up	down	down	N	N
*CiWRKY3–2*	up	down	N	up	N
*CiWRKY6–1*	up	down	up	down	N
*CiWRKY6–2*	up	up	up	up	up
*CiWRKY12*	up	down	up	down	down
*CiWRKY15*	down	down	up	up	up
*CiWRKY17*	up	down	up	down	down
*CiWRKY23*	up	N	up	N	N
*CiWRKY26*	up	up	up	up	up
*CiWRKY28–1*	up	down	up	down	down
*CiWRKY28–2*	up	N	up	down	N
*CiWRKY30*	up	down	up	up	up
*CiWRKY32–1*	N	down	N	N	down
*CiWRKY33–1*	down	down	down	up	up
*CiWRKY36*	up	down	up	down	N
*CiWRKY40–1*	up	up	up	up	up
*CiWRKY40–3*	up	down	up	up	N
*CiWRKY40–4*	up	up	up	up	up
*CiWRKY41–1*	up	N	up	up	up
*CiWRKY48*	up	down	up	down	down
*CiWRKY50*	up	down	up	down	up
*CiWRKY51*	up	down	up	down	up
*CiWRKY56*	up	down	up	down	down
*CiWRKY57*	up	down	up	up	N
*CiWRKY69–1*	down	down	N	up	N
*CiWRKY70–1*	up	N	up	up	down
*CiWRKY75–1*	up	up	up	up	up

One-month-old *C. intermedia* seedlings were subjected to drought, salt, ABA, cold and high-pH treatments, and shoot samples were collected at 0.5, 1, 3, 6, 12, 24 or 48 h. Untreated plants were employed as controls. “Up” indicates up-regulated expression with a minimum 2-fold change compared with the control. “Down” indicates down-regulated expression with a minimum 2-fold change compared with the control. “N” indicates no obvious change detected

### *CiWRKY75–1* transgenic Arabidopsis is hypersensitive to drought stress

**Fig. 6:**
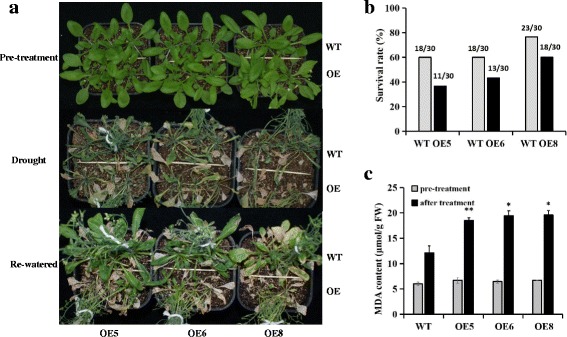
Transgenic *Arabidopsis* overexpressing *CiWRKY75–1* is hypersensitive to drought stress. Four-week-old *CiWRKY75–1* transgenic and wild-type *Arabidopsis* plants were subjected to drought stress by withholding water for 15 days, followed by re-watering for 2 days. **a** Photograph of representative plants. **b** Calculation of survival rates. Thirty plants from each genotype were used for statistical analysis. **c** Measurement of MDA contents. * indicates significant differences (*P* < 0.05); ** indicates extremely significant differences (*P* < 0.01). OE represents overexpression lines, WT represents wild-type *Arabidopsis*. The experiments were repeated three times with similar results

*CiWRKY75–1*, which exhibited relatively high expression under all five treatments (Table 2), was over-expressed in *Arabidopsis*. Three representative overexpression lines (*CiWRKY75–1-OE5*, *CiWRKY75–1-OE6* and *CiWRKY75–1-OE8*) showing relatively high expression levels were used to perform abiotic stress tolerance tests. Four-week-old transgenic *Arabidopsis* were exposed to drought stress by withholding watering for 15 days and were then re-watered for 3 days. The *CiWRKY75–1*-overexpressing lines were more sensitive to drought, displaying decreased survival rates and significantly increased MDA levels compared with the wild type (Fig. 6). No morphological differences were observed between the wild-type and *CiWRKY75–1*-overexpressing plants under normal growth conditions (Fig. 6). These results indicated that overexpression of *CiWRKY75–1* reduced the tolerance of *Arabidopsis* to drought stress, and CiWRKY75–1 acted as a negative regulator in the plant response to drought.

### *CiWRKY40–4-*overexpressing lines show delayed leaf senescence

**Fig. 7:**
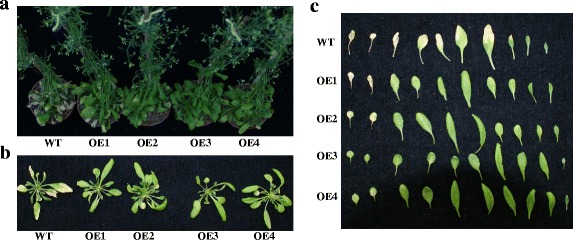
Overexpression of *CiWRKY40–4* delayed *Arabidopsis* leaf senescence. Six-week-old *CiWRKY40–4*-overexpressing lines growing in normal conditions exhibited a stay-green phenotype compared with wild-type *Arabidopsis*. OE represents overexpression lines, WT represents wild-type *Arabidopsis*. **a** The adult plants of different genotypes growing under normal conditions. **b** The rosette leaves of the plants from **a**. **c** The detached rosette leaves from **b**

**Fig. 8:**
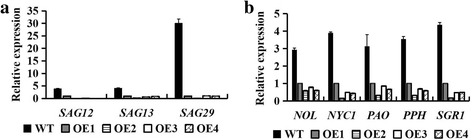
Expression patterns of senescence-related genes in *CiWRKY40–4*-overexpressing and wild-type *Arabidopsis*. Leaf samples were collected from five-week-old *CiWRKY40–4*-overexpressing lines and wild-type *Arabidopsis* and were employed for expression analyses through quantitative real-time PCR. **a** Expression patterns of senescence-associated genes (*SAG12*, *SAG13* and *SAG29*). **b** Expression patterns of chlorophyll degradation-related genes (*PPH*, *PAO*, *SGR1/NYE1*, *NOL* and *NYC1*). Expression values were estimated using the 2^-ΔΔCT^ method, and *AtEF1α* was used as the reference gene. The error bars represent the mean of three technical replicates ± SD. The experiments were repeated three times with similar results

According to the observed stress-responsive expression patterns, *CiWRKY40–4* was another gene that was induced under all five treatments (Table 2). However, ectopic expression of *CiWRKY40–4* in *Arabidopsis* resulted in no obvious morphological differences from the wild type under drought stress. Unexpectedly, under normal growth conditions, *CiWRKY40–4*-overexpressing *Arabidopsis* showed delayed leaf senescence compared with the wild type (Fig. 7). To further explore the mechanism of CiWRKY40–4 in leaf senescence, senescence related-genes, including the senescence-associated genes *SAG12*, *SAG13* and *SAG29* and the chlorophyll degradation-related genes *pheophytinase* (*PPH*), *pheophorbide a oxygenase* (*PAO*), *non-yellowing1*/*stay-green1* (*NYE1/SGR1*), *non-yellow coloring1* (*NYC1*) and *NYC1-like* (*NOL*) were analyzed. All of these genes were down-regulated in the *CiWRKY40–4*-overexpressing lines compared with the wild type (Fig. 8), indicating that CiWRKY40–4 delayed plant senescence by negatively regulating senescence-associated gene expression in *Arabidopsis*.

## Discussion

In this study, we identified 53 CiWRKY-encoding genes from the drought-treated transcriptome database (NCBI Sequence Read Achieve (SRA) accession number SRP121096) based on the presence of WRKY domains [3]. According to the number of their WRKY domains and the features of their zinc finger-like motifs, the CiWRKYs were divided into three groups, as generally described, among which the group II CiWRKYs accounted for the largest proportion of 64%. These findings are consistent with reports regarding the WRKYs found in *A. thaliana* [3], cassava [11], and soybean [7], which harbor the greatest numbers of group II WRKYs in the family.

Generally, WRKY proteins are defined by the conserved amino acid sequence WRKYGQK [3]; however, this sequence has been replaced by WRRY, WSKY, WKRY, WVKY or WKKY in some WRKY proteins [5]. In the present study, the WKKYEEK variant was observed in CiWRKY44–1 proteins, and the WRKYGKK variant motif replaced the conserved WRKYGQK sequence in CiWRKY41–2, 50 and 51. These findings are consistent with reports describing the WRKY proteins identified in other species, such as *A. thaliana* (AtWRKY51, 52 and 59) [3], *V. vinifera L.* (VvWRKY8, 13, 14 and 24) [53], and *P. miliaceum L.* (PmWRKY2, 15, 23, 24, and 28) [14].

According to recent reports [54], several of the WRKYs have more than 2 WRKY domains or contain other domains, in addition to the WRKY domains. These other domains include, for example, the ZF_SBP TF domain, kinase domain, PAH domain, ULP_protease domain, TIR domain, LRR domains, NAC domain, ATP_GRASP domain, and B3 domain. Based on these findings, additional novel groups, beyond the three known WRKY groups, were identified [54]. In *A. thaliana*, *Fragaria vesca*, *Brassica rapa*, *G. max*, *G. raimondii*, and *Sorghum bicolor,* 1, 5, 3, 1, 2, and 3 novel WRKY proteins have been found, respectively [54]. We report here that all of the 28 identified CiWRKYs with ORFs contained one or two WRKY domains. In addition to the WRKY domains, NCBI conserved domain database (CDD) annotation [55] showed that CiWRKY36 (Group IIb) contains a DUF972 (domain of unidentified function 972) domain, followed by a WRKY domain, and both CiWRKY15 and CiWRKY17 (Group IId) harbor a plant_Zn_cluster domain, which is located at the N-terminus and is followed by a WRKY domain. CiWRKY6–1 (Group IIb) exhibits a bZIP (basic region/leucine zipper) domain, followed by a WRKY domain. DUF972 belongs to a protein family of unknown function. The plant_Zn_cluster domain is present in two CiWRKYs (CiWRKY15 and CiWRKY17) and is located at the N-terminus of the WRKY domain. Its sequence (EHSDDVSGSGKCHCVKRRKNRVKRTVRVPAISSKIAD) is different from the zinc finger structure, and this domain may have additional functions. bZIP transcription factors are also one of the largest gene families in plants and are involved in multiple plant biological processes, including various stress responses [56, 57].

Evidence from genome- or transcriptome-wide analyses shows that the response of *WRKYs* to abiotic stresses varies according to the species. For instance, 30 putative *WRKYs* were identified through transcriptome analysis in Chinese wild hazel (*Corylus heterophylla Fisch*), and 23 of these *WRKYs* were found to be simultaneously up-regulated by cold, drought and high-salinity stresses [58]. Twenty-eight salt-responsive *GarWRKYs* were identified among 109 *WRKY* genes in a salt-tolerant wild cotton species (*Gossypium aridum*) based on transcriptome sequencing data [59]. In *P. miliaceum L.*, 32 *WRKYs* were identified based on transcriptome analysis, and the expression levels of 22 *PmWRKYs* were observed to be significantly altered under at least one abiotic stress treatment [14]. Among the 85 *WRKY* genes identified from the *M. esculenta* genome, 78 were found to be differentially expressed in response to drought stress [11]. In addition, 34 out of 127 *WRKYs* from the apple genome were identified as differentially expressed under water-logging stress [8].

Drought is one of the most common environmental stress factors that significantly inhibits plant growth and crop production [60]. The roles of the WRKY transcription factors in the drought response have been widely reported. Activated or constitutive expression of *WRKY57* confers drought tolerance in *A. thaliana* [34], and the expression of *AtWRKY57* is increased by abiotic stresses such as ABA, dehydration, and mannitol treatment [34]. Overexpression of *AtWRKY53* results in hypersensitivity to drought stress by inhibiting stomatal closure [61]. Overexpression of *TaWRKY1* and *TaWRKY33* in *Arabidopsis* leads to enhanced plant drought tolerance [62]. *WRKY20* transcripts accumulate in wild soybean (*G. soja*) under ABA, salt, cold, and drought stresses, and overexpression of *GsWRKY20* increases the drought tolerance in *A. thaliana* [33].

We performed a transcriptome analysis to profile gene expression patterns in *C. intermedia* in response to drought stress and identified 53 *CiWRKY* genes with varying expression patterns in response to drought stress (Additional file 7: Table S2). Further investigation showed that the transcripts of 28 *CiWRKYs* (with complete ORFs) responded to at least one abiotic stress when plants were subjected to drought, cold, salt, high-pH, and ABA treatments. The expression levels of fifteen genes (*CiWRKY12*, *17*, *15*, *28–1*, *30*, *33–1*, *40–1*, *50*, *51*, *48*, *56*, *26*, *75–1*, *40–4*, and *6–2*) were altered under all five treatments. Notably, *CiWRKY40–1* and *CiWRKY75–1* responded to all treatments intensively. In *A. thaliana*, AtWRKY75, which is a homologue of CiWRKY75–1 sharing 85% amino acid sequence similarity, has been reported to regulate Pi starvation and root development [63] and to play a role in plant resistance to pathogens [64, 65]. In the present study, the overexpression of *CiWRKY75–1* in *Arabidopsis* compromised the drought stress tolerance of plants. This finding has not been reported by other researchers, and suggests that CiWRKY75–1 serves as a negative regulator under drought stress. Both the mRNA and protein of AtWRKY53 are reported to accumulate in *Arabidopsis* following drought stress, and *AtWRKY53* overexpression enhances hypersensitivity to drought stress compared with that in the wild type, via inhibition of stomatal closure to increase the water loss rate [61]. The *AtWRKY46* transcript is also induced by drought stress in *Arabidopsis* [66], and the AtWRKY46/54/70 signaling complex negatively regulates plant drought tolerance by repressing dehydration-inducible gene expression, which is likely achieved via the simultaneous function of BES1 [67]. However, all four *Arabidopsis* WRKYs belong to group III, while CiWRKY75–1 belongs to group IIc; thus, the mechanism underlying the regulation of the drought tolerance signaling pathway by CiWRKY75–1, either through direct or indirect interaction with downstream genes, remains unclear. Additionally, no morphological differences during root development were observed between the overexpression lines and wild-type *Arabidopsis*, and the functions of CiWRKY75–1 in pathogen infection and Pi starvation still require further investigation.

CiWRKY40–4 is in the same group IIa clade as WRKY40, WRKY18, and WRKY60 of *A. thaliana* and is most similar to AtWRKY40. In *A. thaliana,* WRKY18, WRKY40 and WRKY60 play partially redundant roles in response to *P. syringae* and *B. cinerea* infection [68]. The *wrky18wrky40* double mutant displays enhanced resistance towards the powdery mildew *Golovinomyces orontii*. This mutant is also involved in PAMP-triggered basal defense and acts as a negative regulator of positive defense regulators such as *CYP71A13*, *EDS1* and *PAD4* [69]. In addition, the expression of *WRKY40*, *WRKY18*, and *WRKY60* is elevated under ABA, NaCl and PEG treatments [44]. The *wrky40* knockout mutant of *A. thaliana* exhibits a sensitive phenotype, while *WRKY40*-overexpressing lines show a less sensitive phenotype than the wild type under ABA-induced inhibition of seed germination [44]. WRKY40 represses the ABA-responsive genes, such as *ABI5*, and functions as negative regulator of in ABA signaling by interacting with ABAR [70]. AtWRKY40 acts as a negative regulator of pathogen-induced cell death regulated by NAC4 [71]. The transcription of *WRKY40*, *WRKY46*, *WRKY51*, *WRKY60*, *WRKY63*, and *WRKY75* is elevated in a loss-of-function mutant of *FtSH4*, which encodes a mitochondrial protease that is involved in SA-mediated leaf senescence [72]. In the present study, overexpressing *CiWRKY40–4* in *A. thaliana* did not alter drought tolerance but delayed leaf senescence by down-regulating the expression levels of senescence-related genes. Senescence is the final step in leaf development and allows nutrients from old leaves to be salvaged [73]. *SAGs*, which are markers of senescence, are up-regulated in the senescing leaves of *A. thaliana* [74]. Overexpression of *SAG29* has been shown to result in accelerated senescence, and osmotic stresses, such as high salinity, cold, and drought, induce the accumulation of its transcripts [75].

*NYC1* and *NOL* encode chlorophyll *b* reductase, which is involved in the biochemical pathway of chlorophyll degradation [76] and accelerates the reduction reaction of chlorophyll *b* in conversion to chlorophyll *a* [73, 76]. PPH is an essential enzyme for chlorophyll breakdown that catalyzes the transformation of pheophytin *a* (Mg-free chlorophyll *a*) into pheophorbide *a* [77], which is then converted to a red chlorophyll catabolite by PAO [78]. The activity of PAO is modulated by NYE1, which is also known as SGR1 [79, 80]. In *A. thaliana*, a *PPH*-deficient mutant (*pph-1*) exhibits a stay-green phenotype due to the prevention of chlorophyll degradation during senescence [77]. The *Atnye1*, *Atpao1*, *Osnol1* and *Osnyc1* mutants all exhibit a stay-green phenotype under natural and/or induction conditions [73, 76, 79, 81]. The down-regulation of *SAGs* (12, 13 and 29), *NYC1*, *NOL*, *PPH*, *PAO* and *NYE1/SGR1*, in *CiWRKY40–4*-overexpressing *Arabidopsis* indicates that CiWRKY40–4 delays *Arabidopsis* leaf senescence by directly or indirectly down-regulating these genes; however, further investigation will be required to elucidate the mechanism.

Taken together, these results indicate that different CiWRKYs play different roles in plant responses to abiotic stress and developmental processes. Hence, there is an urgent need for examination of the function of CiWRKYs in tolerance against abiotic stresses in *C. intermedia* in future studies.

## Conclusions

In this study, 53 *WRKY* sequences were retrieved from the drought-treated transcriptome of *C. intermedia*, among which 28 *CiWRKYs* exhibited full-length sequences. This is the first study to analyze WRKY family genes in *C. intermedia* based on transcriptome data. The predicted proteins were grouped via phylogenetic tree analysis with WRKYs from *A. thaliana* and *G. max*, and their WRKY domains were characterized through multiple sequence alignment of the *CiWRKY* genes. Furthermore, the subcellular localization of both CiWRKY28–1, with one WRKY domain, and CiWRKY26, with two WRKY domains, is nuclear. The majority of the 28 *CiWRKYs* with full-length sequences are expressed in more than one tissue. The expression patterns of the 28 *CiWRKYs* in response to different abiotic stresses (drought, salt, cold, high-pH and ABA) were examined, and the abiotic stress-responsive genes were further evaluated. Additionally, overexpression of *CiWRKY75–1* and *CiWRKY40–4* in *A. thaliana* resulted in hypersensitivity to drought stress and delayed leaf senescence, respectively, compared with the wild type. The results of this study will be useful for understanding the involvement of *WRKY* genes in stress resistance and plant development and will provide the basis for future functional studies on *WRKY*s.

## Methods

### WRKY identification, phylogenetic analysis and multiple sequence alignment

*CiWRKY* sequences were derived from a drought-treated RNA-seq database of *C. intermedia,* in which dehydration treatment was performed by placing the whole seedlings on filter paper for 1, 3 or 12 h. The dataset was submitted to NCBI SRA under the accession number SRP121096. The *WRKY* sequences of *A. thaliana* and *G. max* were downloaded from the TAIR database (The Arabidopsis Information Resource: http://www.arabidopsis.org/) and the JGI database (https://phytozome.jgi.doe.gov), respectively.

The conserved protein domains were subjected to Blast searches against the NCBI database (https://blast.ncbi.nlm.nih.gov/Blast.cgi?PROGRAM=blastx&PAGE_TYPE=BlastSearch&LINK_LOC=blasthome) using blastx. After the removal of redundant sequences and WRKY domain prediction, a total of 53 putative CiWRKYs containing the WRKY domain were obtained, which were named according to their similarity with *Arabidopsis* WRKYs.

A phylogenetic tree of CiWRKYs was constructed using MEGA6.0 and the neighbor-joining (NJ) method, with 1000 bootstrap replications, based on the amino acid sequences of *A. thaliana* and *G. max* WRKY proteins.

DNAMAN7 software was used to analyze the core sequences of the WRKY domain via multiple sequence alignment.

### Plant materials and abiotic stress treatments

Seeds from wild *C. intermedia* were collected from Wulanchabu City, Inner Mongolia Autonomous Region, China (41.44N, 111.69E). No specific field permissions were required to collect the plant samples. The plant material used in this study had been formally identified by Dr. Liwang Qi (Chinese Academy of Forestry), Prof. Ling Yan (Inner Mongolia Agricultural University) and Prof. Meng Ji (Inner Mongolia Academy of Forestry).

The seeds were sown in pots containing peat soil and vermiculite (1:2 *v*/v) under long-day conditions (16-h-light/8-h-dark cycle) at 22 °C. One-month-old plants were employed for the abiotic stress treatments, as previously described [51, 82]. Briefly, prior to the drought, salt, high-pH (10) and ABA treatments, whole seedlings were removed from soil and cleaned with tap water. For drought treatment, whole seedlings were placed on filter paper for 0.5, 1, 3, 6, 12, 24 or 48 h at room temperature. For the salt, high-pH (10) and ABA treatments, the roots of the seedlings were soaked with solutions containing 300 mM NaCl, 200 mM NaHCO_3_ (using NaOH to adjust the pH to 10) or 100 μM ABA, respectively, for 0.5, 1, 3, 6, 12, 24 or 48 h. For cold treatment, pots containing seedlings were transferred to 4 °C and then cultured for 0.5, 1, 3, 6, 12, 24 or 48 h. Untreated plants were used as controls. At each time point in each treatment, the shoots of three plants were harvested as one sample; each plant was only used once for tissue harvesting, at one time point, and was not subjected to any further treatment thereafter. Samples were snap frozen in liquid nitrogen and stored at − 80 °C until they were employed for total RNA extraction. All experiments were repeated three times.

Additionally, the roots, stems, and leaves of wild *C. intermedia* were collected during the flowering stage for tissue-specific expression analysis.

The leaves of four-week-old wild-type and transgenic *Arabidopsis* were used for relative gene expression analysis.

### PRO, SS and MDA measurements

One-month-old *C. intermedia* seedlings cultured in growth chambers were subjected to drought treatment, and physiological indices were measured at different time points (0, 1, 3, 6, 12, 24 or 48 h). The contents of PRO, MDA and SS were determined in colorimetric assays, using the ninhydrin coloration, thiobarbituric acid and anthrone methods, respectively [83].

### Total RNA extraction and cDNA synthesis

Total RNA was isolated from samples using the TRIzol regent. For tissue-specific expression, RNA was extracted from different tissues, including the roots, stems, and leaves. For the examination of abiotic stress-responsive expression, RNA was extracted from shoots. For gene expression analysis in *Arabidopsis*, RNA was extracted from leaves. RNA integrity was examined through agarose gel electrophoresis, and RNA purity was determined based on the A_260 nm_/A_280 nm_ and A_260 nm_/A_230 nm_ ratios.

After pretreatment with RNase-free DNase I (Takara, Dalian, China), total RNA (1 μg), was used to synthesize first-strand cDNA employing an M-MLV reverse transcriptase kit (Takara) according to the manufacturer’s instructions. The cDNA was then diluted 16-fold to be employed as a template for qRT-PCR analysis.

### qRT-PCR analysis of gene expression

qRT-PCR was performed using SYBR Premix Ex Taq II (Takara) on a LightCycler 480 Real Time PCR system (Roche, Basel, Switzerland), as previously described [84]. The thermal cycling program was as follows: 95 °C for 30 s, followed by 40 cycles of 95 °C for 5 s, 60 °C for 30 s and 72 °C for 15 s. *CiEF1α* (GenBank: KC679842) [51] was employed as a reference gene to normalize target gene expression levels in the *C. intermedia* samples, and the 2^-ΔΔCT^ and 2^-ΔCT^ methods [85] were used to calculate the relative expression levels of stress-responsive and tissue-specific genes, respectively. *AtEF1α* (At5G60390) [84] was employed as a reference gene for the normalization of target gene expression levels in *Arabidopsis* samples via the 2^-ΔΔCT^ method [85]. Three technical replicates for each reaction were performed. The primers employed for qRT-PCR analysis are listed in Additional file 8: Table S3.

### *CiWRKY* transgenic *A. thaliana*

To generate the recombinant *CiWRKY* overexpression vector, the full-length CDSs of *CiWRKY75–1* and *CiWRKY40–4* were amplified using PCR product from wild *C. intermedia* cDNA and cloned into the expression vector pCanG-HA using the restriction enzymes *Sal*I/*Spe*I and *Sac*I/*Sal*I, respectively.

To generate the recombinant *CiWRKY* and *GFP*-infused vectors, the coding sequences of *CiWRKY26* and *CiWRKY28–1* without the stop codon were amplified using PCR, and the PCR products were then cloned into the N- terminus of the *GFP*-encoding sequence in the pCAMBIA1302 vector driven by the cauliflower mosaic virus (CaMV) 35S promoter. The primers used for the construction of all of the recombination vectors are listed in Additional file 8: Table S3.

The recombinant vectors were expressed in wild-type *A. thaliana* using the floral dipping method [86], mediated by *Agrobacterium tumefaciens* (strain GV3101). The empty vector was used as the control.

### Subcellular localization of CiWRKYs

The roots of 10-day-old T_2_-generation transgenic seedlings were employed for observations of GFP fluorescence under an LSM510 confocal laser-scanning microscope (Carl Zeiss), with 488 nm argon excitation and a 505–530 nm band filter, as previously described [84].

### Drought tolerance test

To assess the potential drought tolerance of the *CiWRKY*-overexpressing lines, four-week-old transgenic plants and wild-type plants growing in the same pots were exposed to drought stress by withholding water for 15 days, and the plants were then re-watered and allowed to grow for an additional 3 days. Survival rates were calculated using 30 plants per genotype for each experiment. The leaf samples were harvested before or immediately after drought treatment to measure physiological indices. This experiment was repeated three times with similar results.

## Additional files

Additional file 1: Table S1. Expression data for *CiWRKY* genes in different *C. intermedia* tissues. The expression data were generated via qRT-PCR, and calculations were performed using the 2^-ΔCT^ method; *CiEF1α* was used as an endogenous control. (XLS 22 kb)

Additional file 2: Figure S1. Changes in physiological activity in *C. intermedia* under drought treatment. The abscissa indicates the time points in the treatments, and the ordinate indicates physiological activities. (a) MDA content. (b) Pro content. (c) SS content. (PDF 112 kb)

Additional file 3: Figure S2. Expression patterns of *CiWRKYs* under salt treatment. Samples were collected from the shoots of one-month-old *C. intermedia* seedlings at 0.5, 1, 3, 6, 12, 24 or 48 h following salt treatment, and untreated plants were employed as the control. The expression levels of 28 *CiWRKYs* with full-length sequences were examined via qRT-PCR. Expression values were estimated using the 2^-ΔΔCT^ method, and *CiEF1α* was used as reference gene. The error bars represent the means of three technical replicates ± SD. (PDF 258 kb)

Additional file 4: Figure S3. Expression patterns of *CiWRKYs* under ABA treatment. Samples were collected from the shoots of one-month-old *C. intermedia* seedlings at 0.5, 1, 3, 6, 12, 24 or 48 h following ABA treatment, and untreated plants were employed as the control. The expression levels of 28 *CiWRKYs* with full-length sequences were examined via qRT-PCR. Expression values were estimated using the 2^-ΔΔCT^ method, and *CiEF1α* was used as reference gene. The error bars represent the means of three technical replicates ± SD. (PDF 254 kb)

Additional file 5: Figure S4. Expression patterns of *CiWRKYs* under cold treatment. Samples were collected from the shoots of one-month-old *C. intermedia* seedlings at 0.5, 1, 3, 6, 12, 24 or 48 h following cold treatment, and untreated plants were employed as the control. The expression levels of 28 *CiWRKYs* with full-length sequences were examined via qRT-PCR. Expression values were estimated using the 2^-ΔΔCT^ method, and *CiEF1α* was used as reference gene. The error bars represent the means of three technical replicates ± SD. (PDF 253 kb)

Additional file 6: Figure S5. Expression patterns of *CiWRKYs* under high-pH treatment. Samples were collected from the shoots of one-month-old *C. intermedia* seedlings at 0.5, 1, 3, 6, 12, 24 or 48 h following high-pH treatment, and untreated plants were employed as the control. The expression levels of 28 *CiWRKYs* with full-length sequences were examined via qRT-PCR. Expression values were estimated using the 2^-ΔΔCT^ method, and *CiEF1α* was used as reference gene. The error bars represent the means of three technical replicates ± SD. (PDF 257 kb)

Additional file 7: Table S2. Expression data for 28 *CiWRKY* genes with complete ORFs based on drought-treated transcriptome data. (XLS 22 kb)

Additional file 8: Table S3. Primers employed for qRT-PCR and the analysis of transgenic plant construction. (XLS 27 kb)

## Abbreviations

ABA: Abscisic acid
GFP: Green fluorescent protein
MDA: Malondialdehyde
*NOL*: *NYC1-like*
*NYC1*: *Non-yellow coloring1*
*NYE1/SGR1*: *Non-yellowing1*/*stay-green1*
OE: Overexpression
ORF: Open reading frame
*PAO*: *Pheophorbide a oxygenase*
*PPH*: *Pheophytinase*
PRO: Proline
qRT-PCR: Quantitative real-time PCR
RACE: Rapid-amplification of *cDNA* ends
SS: Soluble total sugar
