# Supplementary figures and images for: Identification of the WRKY gene family and functional analysis of two genes in Caragana intermedia
Source: BMC Plant Biol. 2018 Feb 9;18:31. doi: 10.1186/s12870-018-1235-3 (PMC5807834; doi:10.1186/s12870-018-1235-3)

**a**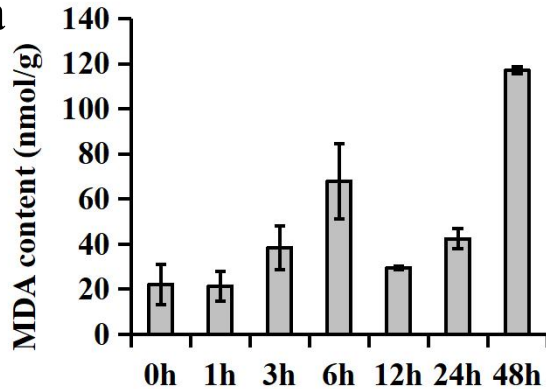**b**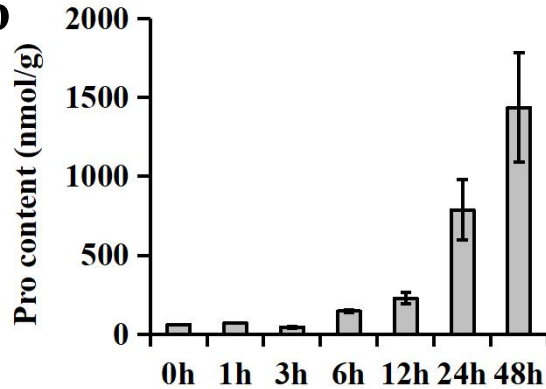**c**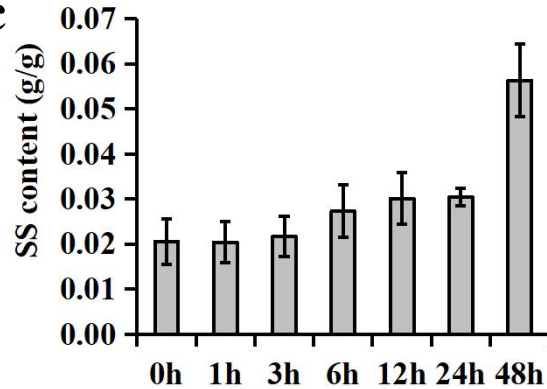

Supplement: Supplementary file 2 — Changes in physiological activity in C. intermedia under drought treatment. The abscissa indicates the time points in the treatments, and the ordinate indicates physiological activities. (a) MDA content. (b) Pro content. (c) SS content. (PDF 112 kb) [file 12870_2018_1235_MOESM2_ESM.pdf]

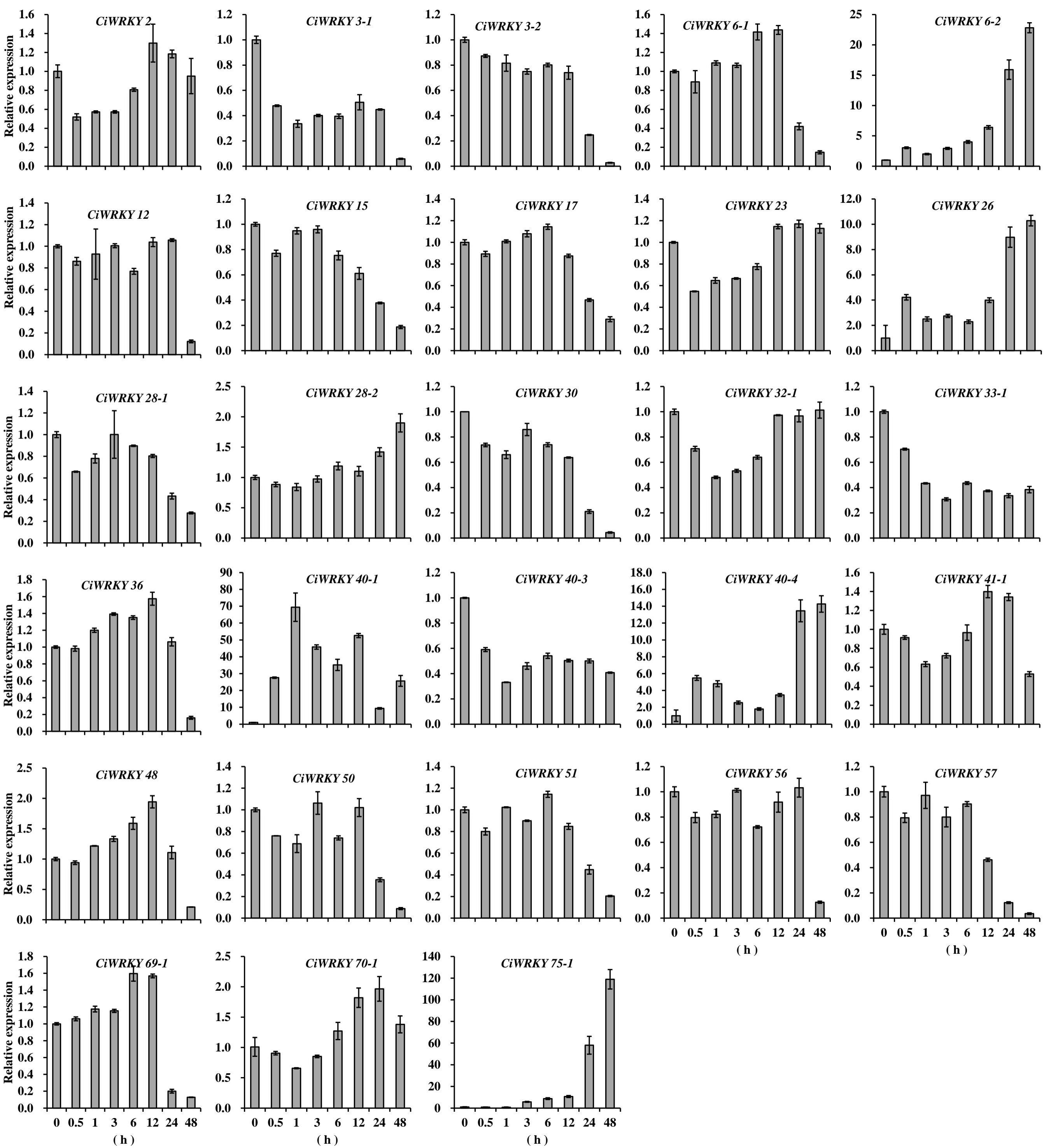

Supplement: Supplementary file 3 — Expression patterns of CiWRKYs under salt treatment. Samples were collected from the shoots of one-month-old C. intermedia seedlings at 0.5, 1, 3, 6, 12, 24 or 48 h following salt treatment, and untreated plants were employed as the control. The expression levels of 28 CiWRKYs with full-length sequences were examined via qRT-PCR. Expression values were estimated using the 2-ΔΔCT method, and CiEF1α was used as reference gene. The error bars represent the means of three technical replicates ± SD. (PDF 258 kb) [file 12870_2018_1235_MOESM3_ESM.pdf]

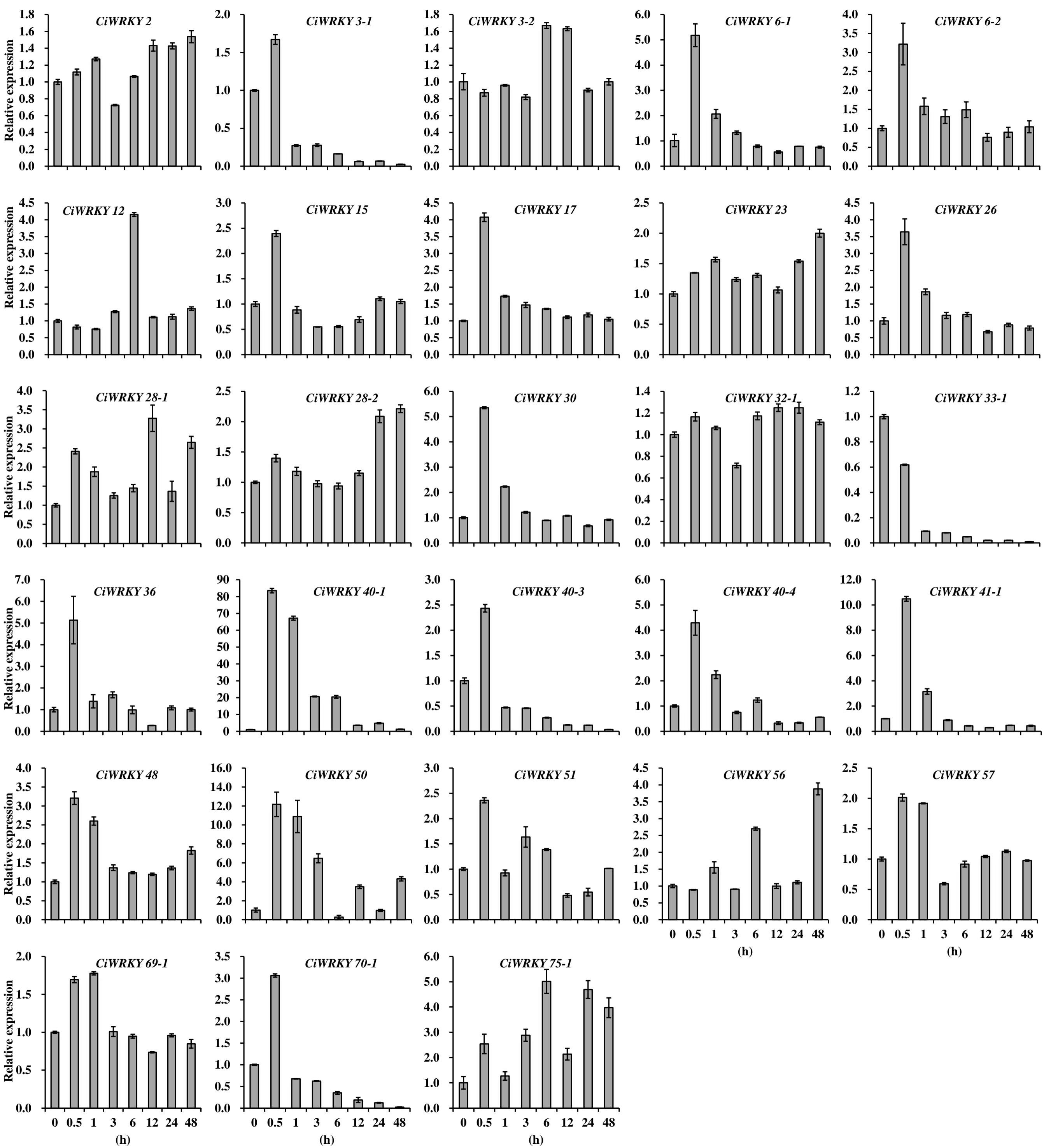

Supplement: Supplementary file 4 — Expression patterns of CiWRKYs under ABA treatment. Samples were collected from the shoots of one-month-old C. intermedia seedlings at 0.5, 1, 3, 6, 12, 24 or 48 h following ABA treatment, and untreated plants were employed as the control. The expression levels of 28 CiWRKYs with full-length sequences were examined via qRT-PCR. Expression values were estimated using the 2-ΔΔCT method, and CiEF1α was used as reference gene. The error bars represent the means of three technical replicates ± SD. (PDF 254 kb) [file 12870_2018_1235_MOESM4_ESM.pdf]

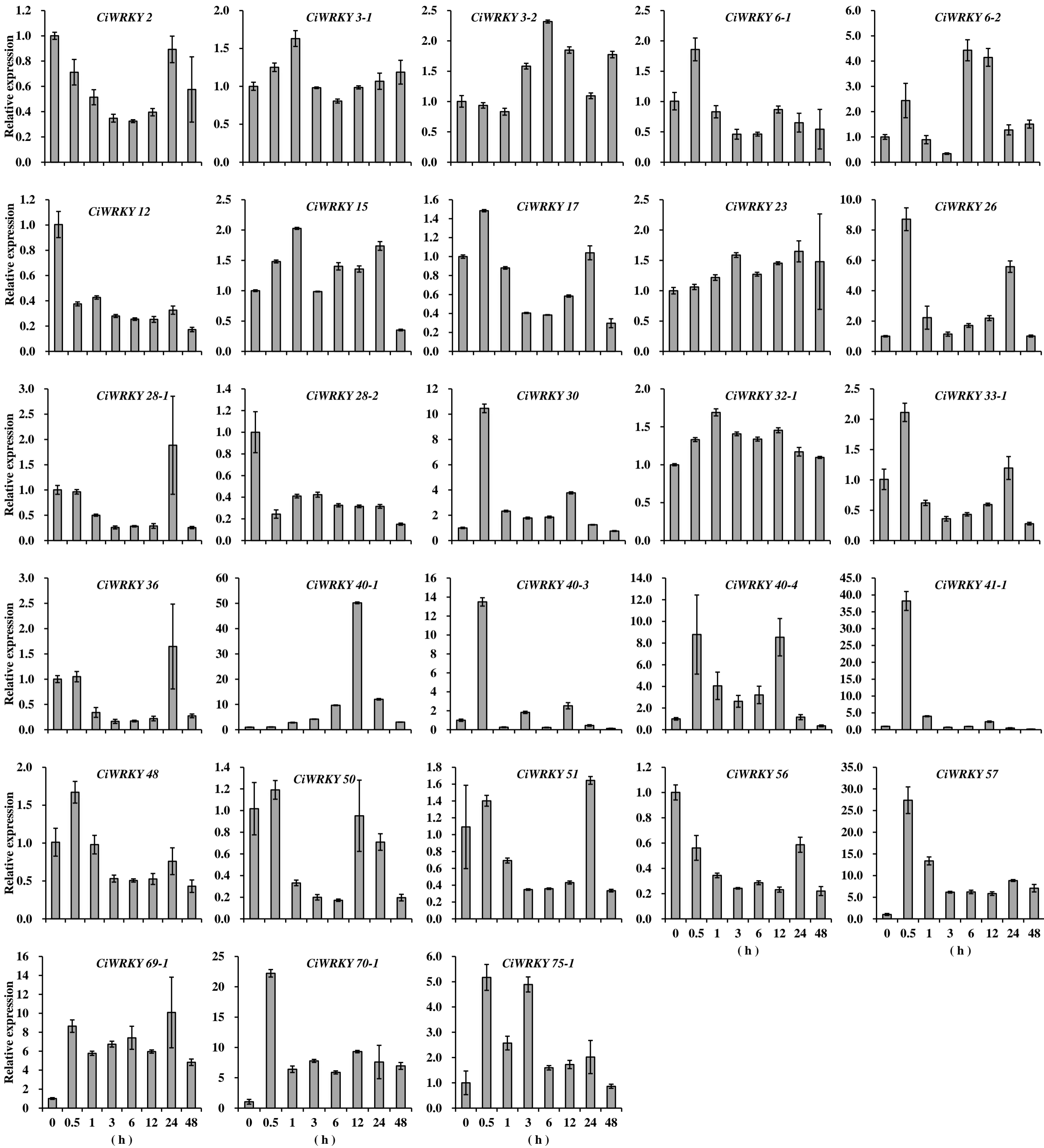

Supplement: Supplementary file 5 — Expression patterns of CiWRKYs under cold treatment. Samples were collected from the shoots of one-month-old C. intermedia seedlings at 0.5, 1, 3, 6, 12, 24 or 48 h following cold treatment, and untreated plants were employed as the control. The expression levels of 28 CiWRKYs with full-length sequences were examined via qRT-PCR. Expression values were estimated using the 2-ΔΔCT method, and CiEF1α was used as reference gene. The error bars represent the means of three technical replicates ± SD. (PDF 253 kb) [file 12870_2018_1235_MOESM5_ESM.pdf]

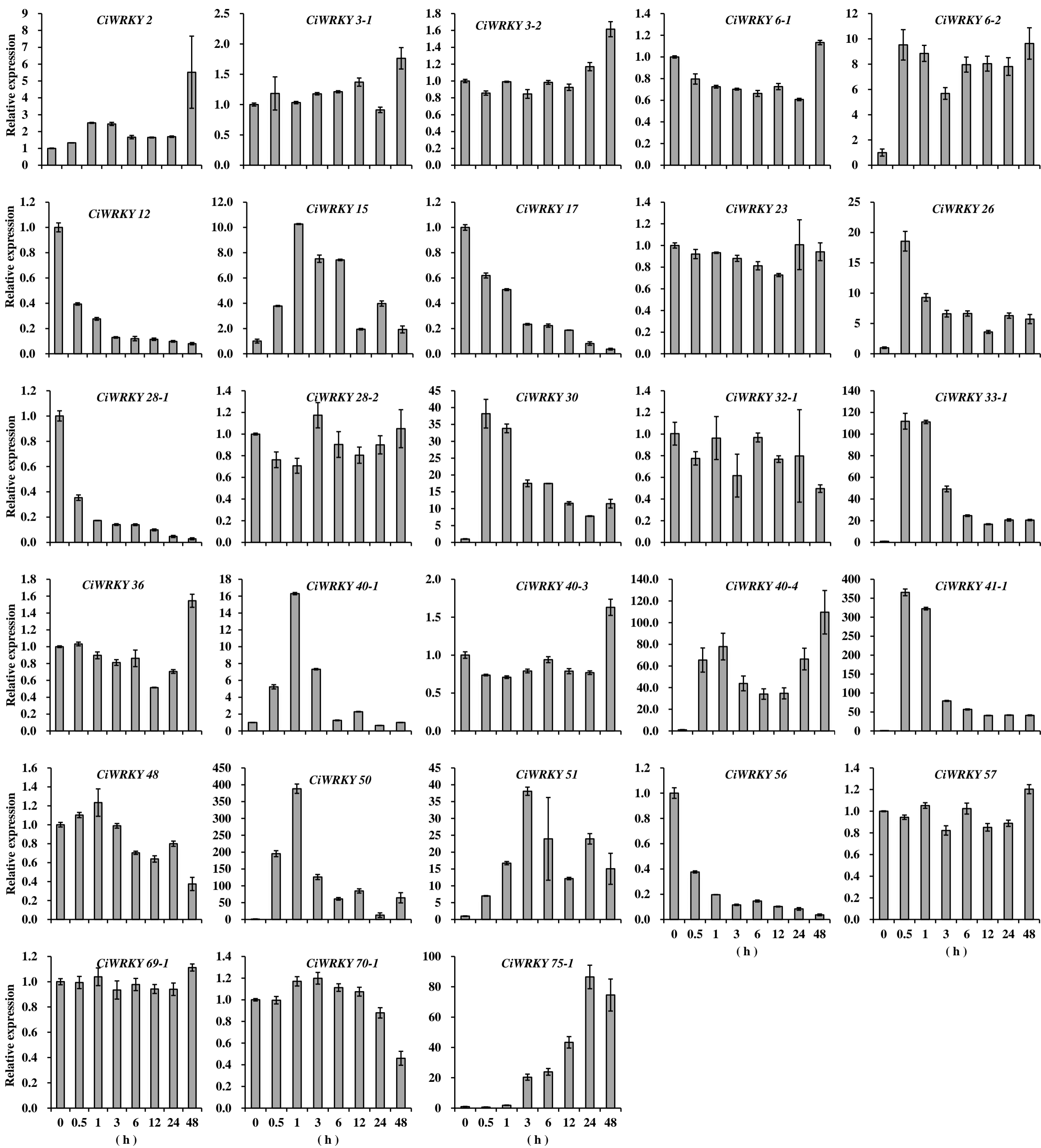

Supplement: Supplementary file 6 — Expression patterns of CiWRKYs under high-pH treatment. Samples were collected from the shoots of one-month-old C. intermedia seedlings at 0.5, 1, 3, 6, 12, 24 or 48 h following high-pH treatment, and untreated plants were employed as the control. The expression levels of 28 CiWRKYs with full-length sequences were examined via qRT-PCR. Expression values were estimated using the 2-ΔΔCT method, and CiEF1α was used as reference gene. The error bars represent the means of three technical replicates ± SD. (PDF 257 kb) [file 12870_2018_1235_MOESM6_ESM.pdf]
